# The novel second-generation triterpenoid SCY-247 maintains *in vitro* and *in vivo* activity against resistant *Candida glabrata*

**DOI:** 10.1128/aac.01625-25

**Published:** 2026-02-19

**Authors:** Nathan P. Wiederhold, Laura K. Najvar, Rosie Jaramillo, Marcos Olivo, Hoja P. Patterson, Thomas F. Patterson

**Affiliations:** 1 University of Texas Health Science Center at San Antonio 14742, San Antonio, Texas, USA; University of Iowa, Iowa City, Iowa, USA

**Keywords:** SCY-247, *Candida glabrata*, triterpenoid, echinocandin resistance, murine model, invasive candidiasis

## Abstract

*Candida glabrata* is a major cause of invasive candidiasis and is considered a high-priority fungal pathogen by the World Health Organization. SCY-247 is a second-generation IV/oral triterpenoid antifungal that targets the fungal cell wall by inhibiting glucan synthase. We evaluated the *in vitro* activity and *in vivo* efficacy of SCY-247 against echinocandin-resistant *C. glabrata*. Susceptibility testing was performed against 34 *C. glabrata* clinical strains, including 29 echinocandin non-susceptible or resistant strains, by the CLSI broth microdilution method. Neutropenic mice were infected intravenously with either an echinocandin-susceptible or resistant strain. Treatment with vehicle control, SCY-247 (16, 32, and 48 mg/kg PO BID), fluconazole (20 mg/kg PO QD), or caspofungin (5 mg/kg IP QD) was initiated 24 hours post-inoculation. Treatment continued for 7 days, and kidney and lung tissues were collected on day 8 for analysis of fungal burden. SCY-247 maintained *in vitro* activity against 24 of the 29 echinocandin non-susceptible/resistant strains; SCY-247 was also efficacious against echinocandin-susceptible and resistant *C. glabrata* invasive candidiasis. Dose-dependent reductions in kidney and lung fungal burdens were observed in mice treated with SCY-247. In contrast, neither fluconazole nor caspofungin led to reductions in fungal burden in mice infected with the resistant strain. SCY-247 concentrations measured 12 hours after the last dose increased in a dose-dependent fashion, and those within the kidneys and lungs were markedly higher than the SCY-247 MIC_90_ value calculated against all strains tested. These data support the potential utility of SCY-247 therapy against invasive infections caused by resistant *C. glabrata*.

## INTRODUCTION

Of the invasive mycoses, invasive candidiasis is often the most prevalent, as *Candida* species are common causes of hospital-acquired pathogens and nosocomial bloodstream infections (1–3). These invasive infections are associated with significant morbidity and mortality (1, 4, 5). In the United States, the second most prevalent species associated with invasive infection is *Candida glabrata* (now known as *Nakaseomyces glabratus*) (3, 4). In 2022, this species was designated as a high-priority fungal pathogen by the World Health Organization (6). Current treatment strategies for invasive infections caused by this pathogen include the use of the azoles (e.g., fluconazole, isavuconazole, voriconazole, and posaconazole), a lipid formulation of amphotericin B (polyene), or the echinocandins (anidulafungin, caspofungin, micafungin, and rezafungin) (7). Although effective, each of these classes has drawbacks that may limit clinical responses. For example, amphotericin B formulations are associated with significant nephrotoxicity and are only available intravenously. The echinocandins, although clinically safer than the polyenes, are also only available for intravenous administration, which may limit their use in outpatients. Fluconazole has been a safe and effective treatment option over many years for patients with invasive candidiasis, and this antifungal is available in both oral and intravenous formulations. However, *C. glabrata* demonstrates reduced susceptibility to fluconazole, and invasive disease caused by fluconazole- and/or echinocandin-resistant *C. glabrata* strains has been documented at many centers in the United States and other parts of the world (8, 9). Thus, the development of new therapeutic strategies for invasive candidiasis, including those infections caused by resistant *C. glabrata,* is of paramount importance.

SCY-247 is a second-generation member of the triterpenoid class of antifungals and has potent *in vitro* activity against *Candida* species, including *C. auris*, other yeasts, and several filamentous fungi (10, 11). This *in vitro* activity has also translated into *in vivo* efficacy in a murine model of invasive candidiasis caused by *C. albicans* (12). The objective of this study was to evaluate the *in vitro* and *in vivo* activity of SCY-247 against *C. glabrata,* including echinocandin-resistant strains.

## RESULTS

### *In vitro* susceptibility

**Fig 1:**
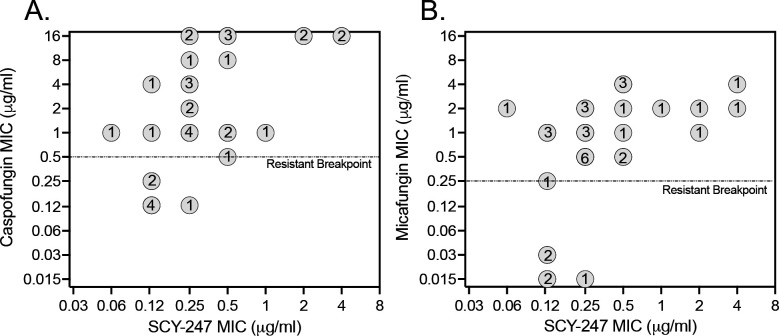
SCY-247 versus caspofungin MICs (**A**) and SCY-247 versus micafungin MICs (**B**) against 29 echinocandin non-susceptible or resistant strains of *Candida glabrata* and 5 echinocandin-susceptible strains. All values are reported in μg/mL. Dotted horizontal lines represent CLSI resistant breakpoints for caspofungin (0.5 μg/mL) and micafungin (0.25 μg/mL) against *Candida glabrata*. Circled numbers represent the number of strains at SCY-247/caspofungin and SCY-247/micafungin MIC combinations. Two strains had caspofungin MIC values that were classified as intermediate (0.25 μg/mL) for this echinocandin.

SCY-247 MICs ranged from 0.06 to 4 μg/mL against all strains (*n* = 34) included in this study (Table 1). SCY-247, caspofungin, and micafungin MICs ranged from 0.125 to 0.25 μg/mL, 0.125 μg/mL, and ≤0.015–0.03 μg/mL, respectively, against the 5 echinocandin-susceptible strains. Against the 29 strains classified as echinocandin non-susceptible or resistant strains per the CLSI breakpoints (13), SCY-247 MICs ranged from 0.06 to 4 μg/mL, with MIC_90_ and modal MIC values of 2 μg/mL and 0.25 μg/mL, respectively, and the *in vitro* activity was maintained against 24 of the 29 strains (MIC ≤0.5 µg/mL; Table 1 and Fig. 1). In contrast, against these non-susceptible or resistant strains, caspofungin and micafungin MICs ranged from 0.25 to >8 µg/mL and 0.25 to 4 μg/mL, respectively, with both having higher MIC_90_ values (>8 µg/mL and 4 μg/mL, respectively) and modal MICs (1 μg/mL and 0.5 μg/mL, respectively) than that of SCY-247. SCY-247, fluconazole, and caspofungin MICs were 0.125 μg/mL, 4 μg/mL, and 0.125 μg/mL, respectively, against the echinocandin-susceptible strain used in the *in vivo* model. Against the echinocandin-resistant strain used to establish invasive disease, the MICs were 0.25 μg/mL, 64 μg/mL, and >8 µg/mL, respectively.

**TABLE 1:** MIC parameters and MIC distributions for SCY-247, caspofungin, and micafungin against 34 *Candida glabrata* clinical strains, including 29 echinocandin non-susceptible or resistant clinical strains^*a*^

Parameter	SCY-247				Caspofungin				Micafungin				
All strains													
Range	0.06–4				0.125–>8				≤0.015–4				
MIC_50_	0.25				2				1				
MIC_90_	2				>8				4				
GM^*b*^ MIC	0.325				1.920				0.635				
Modal MIC	0.25				1				0.5				
Echinocandin non-susceptible or resistant strains													
Range	0.06–4				0.25–>8				0.25–4				
MIC_50_	0.25				4				1				
MIC_90_	2				>8				4				
GM MIC	0.375				3.075				1.154				
Modal MIC	0.25				1				0.5				
MIC distributions													
MIC value	0.015	0.03	0.06	0.12		0.25	0.5	1		2	4	8	≥16
SCY-247			1	8		13	7	1		2	2		
Caspofungin				5		2	1	9		2	4	2	9
Micafungin	3	2				1	8	8		8	4		

^*a*^ All values are reported in μg/mL.

^*b*^ GM, geometric mean.

### Fungal burden versus echinocandin-susceptible strain

**Fig 2:**
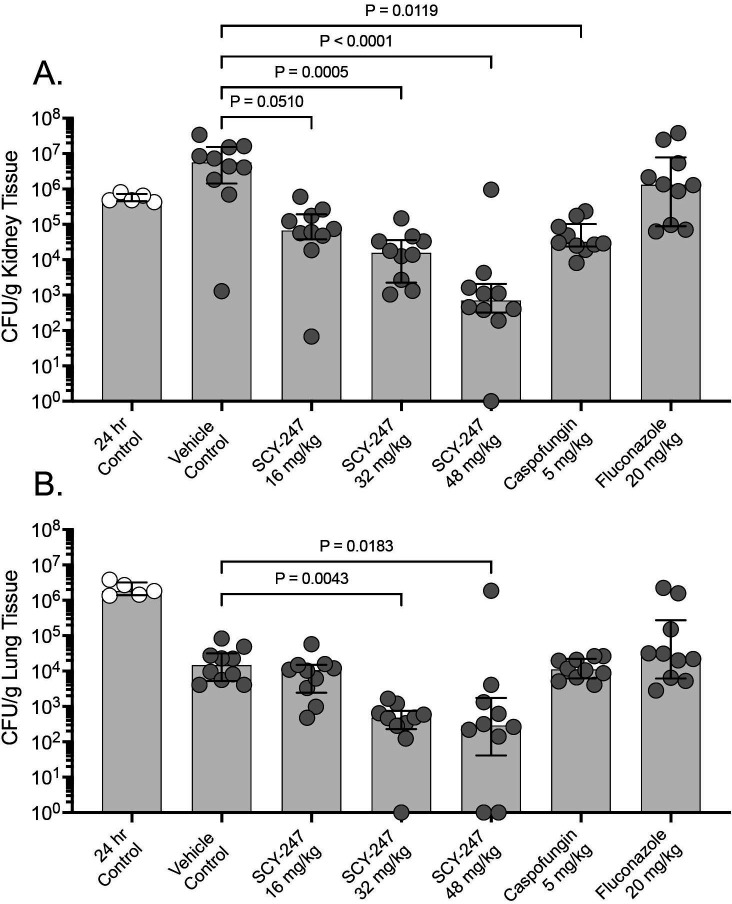
Kidney (**A**) and lung (**B**) fungal burden in mice infected with the echinocandin-susceptible *Candida glabrata* strain at 24 hours post-inoculation, just prior to the start of therapy, and at day 8 in mice treated with vehicle control, SCY-247, caspofungin, or fluconazole by oral gavage for 7 days.

SCY-247 was effective in reducing fungal burden, as measured by CFU counts, in both the kidneys and lungs of mice infected with the echinocandin-susceptible *C. glabrata* strain. A dose-dependent reduction in kidney fungal burden was observed with SCY-247, and CFU counts were significantly lower in mice administered SCY-247 at both the 32 mg/kg and 48 mg/kg dose levels (median values of 4.20 and 2.85 log_10_ CFU/g, respectively) compared to the vehicle control group (6.75 log_10_ CFU/g; *P* ≤ 0.0005 for both comparisons; Fig. 2A). Fungal burden was also numerically lower in mice administered SCY-247 at 16 mg/kg (4.83 log_10_ CFU/g), although this difference did not reach significance compared to the vehicle control group (*P* = 0.0510). Treatment with SCY-247 at doses of 32 mg/kg and 48 mg/kg resulted in reductions in fungal burden of >1 log_10_ CFU/g compared to that measured at 24 hours post-inoculation, just prior to the initiation of treatment, indicating fungicidal activity (Table 2). Fungal burden was also significantly lower in mice that received caspofungin (4.47 log_10_ CFU/g; *P* = 0.0119), with reductions of >1 log_10_ CFU/g compared to that prior to the start of therapy. No reductions in fungal burden were observed with fluconazole (6.12 log_10_ CFU/g). Similar results were also observed in the lungs, where fungal burden was significantly lower in mice that received SCY-247 at 32 mg/kg and 48 mg/kg (median values 2.68 and 2.46 log_10_ CFU/g, respectively) versus vehicle control (4.16 log_10_ CFU/g; *P* ≤ 0.0183 for both comparisons; Fig. 2B). In contrast, no significant reductions in lung fungal burden were observed in mice treated with SCY-247 at 16 mg/kg, caspofungin, or fluconazole compared to the vehicle control group. Marked reductions in lung fungal burden compared to that measured just prior to the start of therapy (24 hours post-inoculation) were observed in all groups, including the vehicle control, consistent with clearance of *Candida* from the lungs.

**TABLE 2:** Changes in fungal burden (log_10_ CFU/g) for each treatment group compared with that prior to the start of therapy (24 hours post-inoculation) against both the echinocandin-susceptible and resistant strains

Treatment group	Echinocandin-susceptible strain		Echinocandin-resistant strain	
	Kidney	Lungs	Kidney	Lungs
Vehicle control	1.07	−2.10	0.19	−1.52
SCY-247 16 mg/kg	−0.86	−2.24	−0.78	−1.67
SCY-247 32 mg/kg	−1.49	−3.58	−1.75	−5.24
SCY-247 48 mg/kg	−2.83	−3.80	−3.70	−6.02
Caspofungin 5 mg/kg	−1.21	−2.22	−0.20	−1.38
Fluconazole 20 mg/kg	0.44	−1.85	0.15	−1.74

### Fungal burden versus echinocandin-resistant strain

**Fig 3:**
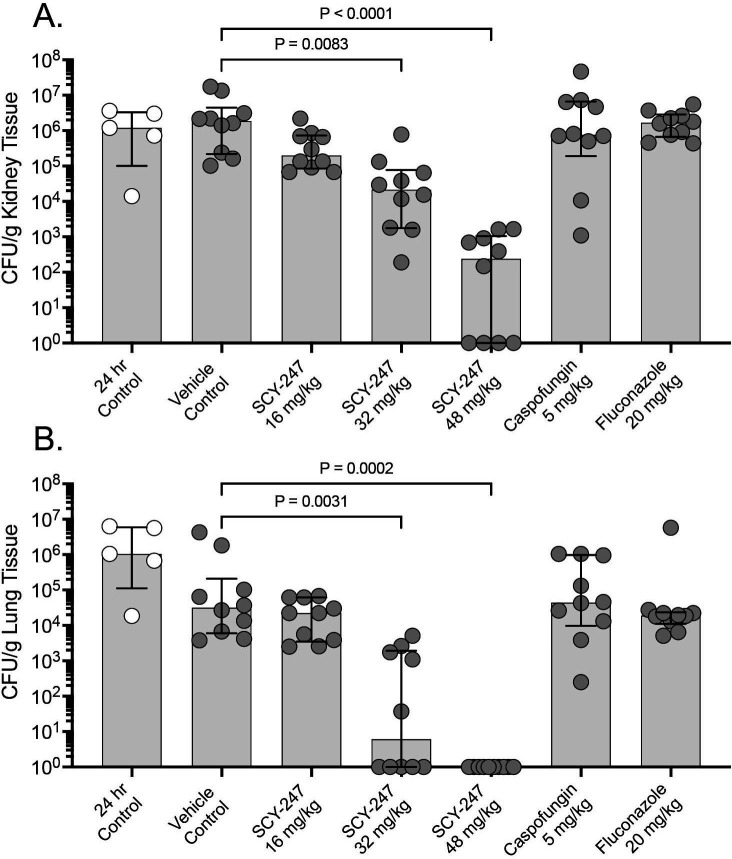
Kidney (**A**) and lung (**B**) fungal burden in mice infected with the echinocandin-resistant *Candida glabrata* strain at 24 hours post-inoculation, just prior to the start of therapy, and on day 8 in mice treated with vehicle control, SCY-247, caspofungin, or fluconazole by oral gavage for 7 days.

SCY-247 was also effective at reducing fungal burden in mice infected with the echinocandin-resistant strain of *C. glabrata*, which harbored the Fks1p S629P mutation, as dose-dependent reductions in CFU counts were observed in mice infected with this strain. Both the 32 mg/kg and 48 mg/kg doses of SCY-247 resulted in significant reductions in kidney fungal burden (4.33 and 2.38 log_10_ CFU/g, respectively) compared to the vehicle control group (6.27 log_10_ CFU/g; *P* ≤ 0.0083 for both comparisons; Fig. 3A). Reductions of greater than 1 log_10_ CFU/g were also observed with these two SCY-247 doses compared to that measured prior to the start of therapy, indicating fungicidal activity against the resistant strain (Table 2). No reductions in kidney fungal burden were observed in the SCY-247 16 mg/kg, caspofungin, or fluconazole groups. Similar results were also observed in the lungs of mice infected with the resistant strain, as both the SCY-247 32 mg/kg and 48 mg/kg dose groups had significantly lower CFU counts (0.78 and 0.00 log_10_ CFU/g, respectively) than the vehicle control (4.50 log_10_ CFU/g; *P* ≤ 0.0031 for each comparison; Fig. 3B). As observed in the kidneys, no reductions in fungal burden were observed in mice treated with a lower dose of SCY-247, caspofungin, or fluconazole. Similar to the echinocandin-susceptible isolate, marked reductions in lung fungal burden compared to that measured just prior to the start of therapy (24 hours post-inoculation) were observed in all groups infected with the echinocandin-resistant strain.

### SCY-247 plasma and tissue concentrations

**Fig 4:**
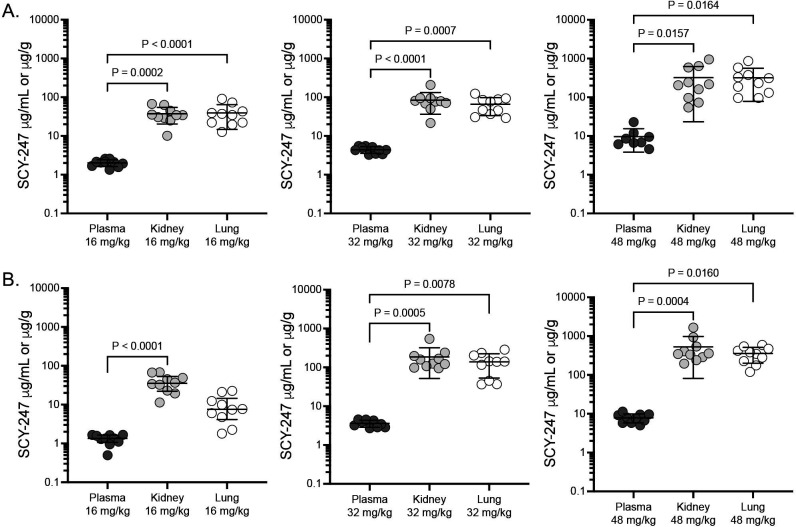
SCY-247 plasma, kidney, and lung concentrations in mice infected with the echinocandin-susceptible strain (**A**) or the echinocandin-resistant strain (**B**) of *Candida glabrata*. Mice were dosed with SCY-247 orally twice daily for 7 days, and plasma, kidneys, and lungs were collected on day 8, 12 hours after the last dose.

SCY-247 trough concentrations were measured in the plasma, kidneys, and lungs of infected mice ~12 hours following the last dose administered on day 7 post-inoculation. SCY-247 concentrations increased in each of these in a dose-dependent fashion (Fig. 4). No differences in achieved concentrations at each dose level were observed between mice infected with the echinocandin-susceptible or echinocandin-resistant strains, although SCY-247 levels were numerically lower in the lungs of mice infected with the resistant strain versus the susceptible strain. The lowest concentrations were observed within the plasma (range of mean plasma concentrations 1.31 to 2.02 μg/mL in the 16 mg/kg group up to 7.62 to 9.63 μg/mL in the 48 mg/kg group). Similar SCY-247 concentrations were observed within the kidneys and lungs at each dose level (range of mean kidney concentrations 38.57 to 530.1 μg/mL; range of mean lung concentrations 9.59 to 355.8 μg/mL), and these were significantly higher than those observed within the plasma (with the exception of the lung concentrations achieved in the 16 mg/kg group in mice infected with the echinocandin-resistant strain). The concentrations achieved within the kidneys and lungs were markedly higher than the SCY-247 MIC against both the susceptible and resistant strains used to establish infection and the MIC_90_ value calculated against all strains tested (MIC_90_, 2 μg/mL).

## DISCUSSION

SCY-247 is an investigational agent that inhibits glucan synthase, leading to a decrease in (1, 3)-β-d-glucan polymers and a weakening of the fungal cell wall (10, 12, 14). Although its mechanism of action is similar to that of the echinocandins, like ibrexafungerp (formerly SCY-078), SCY-247 is structurally different and thus a member of a separate and relatively new class of antifungals, the triterpenoids. In addition, unlike the echinocandins, SCY-247 is absorbed from the gastrointestinal tract following oral administration. In the current study, SCY-247 demonstrated *in vitro* activity against *C. glabrata* isolates that was maintained against the majority of isolates (25 of 29) that were phenotypically resistant or non-susceptible to the echinocandins micafungin and caspofungin. This *in vitro* activity translated into *in vivo* efficacy in a neutropenic murine model of disseminated candidiasis against infections caused by both echinocandin-susceptible and echinocandin-resistant strains in which fungicidal activity was observed with the higher doses of SCY-247 that were used. SCY-247 plasma, kidney, and lung tissue concentrations remained elevated, above the MIC values against both isolates tested, approximately 12 hours after the last doses were administered. Within the kidneys and lungs, the SCY-247 concentrations were also above the MIC_90_ value determined against all strains tested.

The results of the current study are consistent with what others have reported for SCY-247. Previous studies have also demonstrated potent *in vitro* activity for SCY-247 against different *Candida* species, including *C. albicans, C. glabrata,* and *C. auris*, with MIC values similar to those observed for ibrexafungerp (10, 11). Against 65 *C. auris* strains that included members of clades I through V, SCY-247 demonstrated robust activity, with an MIC_50_ of 0.125 μg/mL for isolates that were *FKS* wild-type and 1 μg/mL for those harboring *FKS1* mutants, for which the MICs for anidulafungin and micafungin were markedly elevated (11). *In vitro* activity was also observed against the dimorphic fungi *Coccidioides immitis* and *Histoplasma capsulatum,* as well as *Aspergillus* species (10). *In vitro* activity was also reported against other molds, including *Fusarium* and *Scedosporium* species, but similar to that of ibrexafungerp, the potency of SCY-247 was reduced against these fungi compared to that observed for *Candida* species.

The *in vitro* activity of SCY-247 against *C. albicans* also translated into *in vivo* efficacy in a murine model of disseminated candidiasis. In non-neutropenic mice, treatment with SCY-247 resulted in significant reductions in kidney fungal burden in groups administered doses of 10 mg/kg and 40 mg/kg orally twice daily compared to untreated controls (12). Improvements in survival were also observed, although differences with the untreated control group did not reach statistical significance. Elevated SCY-247 plasma concentrations were also reported after 7 days of therapy. The plasma levels achieved were dose-dependent, and measurable concentrations were noted 36 hours after the last administered dose. The results of this study are consistent with ours in that SCY-247 demonstrated *in vivo* efficacy against invasive candidiasis, with both reductions in fungal burden and achievable concentrations of this investigational agent being dose-dependent.

Overall, the results of our study demonstrate the potential for SCY-247 in the treatment of invasive *C. glabrata* infections, including those caused by echinocandin-resistant strains. However, only one echinocandin-resistant strain was used to establish invasive infection. Thus, additional studies may be needed to confirm our findings, including those in which strains with different FKS mutations and higher SCY-247 MICs are used to establish infection. Additional studies are also needed to understand how different *FKS* mutations, including those not in traditional hotspot regions, may influence SCY-247 activity and whether some cause cross-resistance with ibrexafungerp or the different echinocandins. Finally, while we did measure SCY-247 concentrations at multiple dose levels in different tissues and plasma, we did not include enough dosage groups to conduct a formal pharmacokinetic/pharmacodynamic analysis or demonstrate a clear concentration-response relationship. However, our findings, as well as those reported by others, demonstrate the potential utility of SCY-247 for the treatment of invasive candidiasis.

## MATERIALS AND METHODS

### Antifungals

For *in vitro* susceptibility testing, stock solutions of SCY-247 (Scynexis Inc., Jersey City, NJ, USA), fluconazole, caspofungin, and micafungin (Sigma-Aldrich, St. Louis, MO, USA) were prepared in DMSO. Further dilutions were made in DMSO and then in RPMI (0.165 M MOPS, pH 7.0 without bicarbonate). For the *in vivo* candidiasis model, the acetate salt of SCY-247 (88.7% active moiety SCY-247) was prepared in 0.5% wt/vol methylcellulose in sterile water for injection. Following stirring for 30 minutes, the pH was adjusted to 6.5 using 1 N HCl or 1 N KOH. The preparation was stored protected from light at 2 to 8°C and used within 24 hours of preparation. The clinical intravenous formulations of fluconazole and caspofungin were used for dosing in the *in vivo* model, and the vehicle control was 0.5% methylcellulose.

### *Candida glabrata* isolates

For *in vitro* susceptibility testing, *C. glabrata* clinical strains received by the Fungus Testing Laboratory, UT Health San Antonio, for clinical diagnostic testing were used. Prior to *in vitro* testing, all strains were subcultured twice on Sabouraud dextrose agar (SDA) for 24 hours at 30°C. For the *in vivo* model, echinocandin-susceptible (05-761) and resistant strains (DI24-268) were used. The echinocandin-resistant strain harbored a S629P amino acid substitution in hotspot 1 of Fks1p. These strains were subcultured three times on SDA for 48 hours at 37°C. Cells were collected from the third subculture by scraping the surface of the agar plates. The cells were washed three times in sterile saline with 0.1% Tween 20. After the final wash, an aliquot of cells was collected by centrifugation and resuspended in sterile saline, and a hemocytometer was used to adjust the number of cells to the desired starting inoculum. Viability was confirmed by serial dilution of aliquots onto SDA and counting the number of colonies following inoculation at 37°C.

### *In vitro* susceptibility

*In vitro* susceptibility testing was performed by broth microdilution according to the methods described in the CLSI M27 document (15). The concentration ranges tested were 0.015 to 8 μg/mL for SCY-247, caspofungin, and micafungin, and 0.125 to 64 μg/mL for fluconazole. MICs were read visually after 24 hours of incubation at 35°C as the lowest concentration that resulted in 50% growth inhibition compared to the drug-free control.

### Infection model

We utilized our established murine model of *C. glabrata* invasive candidiasis (16). Briefly, outbred male ICR mice (Envigo, Indianapolis, IN) were housed five per cage and had access to food and water *ad libitum*. Mice were rendered neutropenic with a single dose of 5-fluorouracil (5 mg/mouse) administered intravenously one day prior to infection. On the day of infection (day 0), mice were infected intravenously with 0.2 mL of *C. glabrata* with the target number of *Candida* cells at 1.0 × 10^8^ cells per mouse. Mice were monitored at least twice daily throughout the study to minimize unnecessary pain or distress. One day after inoculation, treatment with vehicle control (methylcellulose by oral gavage twice daily), SCY-247 (16, 32, or 48 mg/kg by oral gavage twice daily), caspofungin (5 mg/kg by intraperitoneal injection once daily), or fluconazole (20 mg/kg by oral gavage once daily) was continued for 7 days. On day 8 post-inoculation, mice were humanely euthanized, and the kidneys and lungs were aseptically removed, weighed, and homogenized. Serial dilutions of the homogenates were plated onto SDA, and after 24 hours of incubation, the number of CFUs per gram of tissue was calculated.

### Measurement of SCY-247 plasma and tissue concentration

SCY-247 plasma, kidney, and lung concentrations were measured in neutropenic, infected mice on day 8, approximately 12 hours following the last administered dose on day 7. Tissue and plasma samples were assayed by LC-MS/MS. All methods met *a priori* method performance acceptance criteria for specificity and inter- and intra-assay performance of <15% coefficient of variation and <±15% accuracy for calibration standards and quality control samples. The calibration range was 5 to 5,000 ng/mL (eight calibration levels). Samples with concentrations beyond this range were diluted. The determination of SCY-247 concentrations in tissues was performed using aliquots of the homogenates prepared for assessment of fungal burden. Mouse plasma (50 µL) or tissue homogenates for standards, quality control, or study samples (50 µL) were treated with 50 µL of internal standard solution (250 ng/mL of SCY-247-d6 in 10:90 dimethyl sulfoxide:acetonitrile) and vortex-mixed for approximately 20 seconds. Sample proteins were precipitated with 1,000 µL of acetonitrile and vortex-mixed for approximately 1 minute. All samples were centrifuged at approximately 14,000 rpm for approximately 5 minutes, whereupon 20 µL were transferred to autosampler vials containing 200 µL of 0.1% (vol/vol) formic acid in 50:50 acetonitrile/water. Analysis was performed employing a Shimadzu (Columbia, MD, USA) HPLC system comprising a SIL-30AC autosampler and an LC-20AD pump and an Applied Biosystems (Foster City, CA, USA) API-5500 QTRAP mass spectrometer operated as a triple quadrupole instrument with a turbo-ion electrospray source in positive ionization mode.

### Data analysis

Descriptive statistics were used for the *in vitro* susceptibility results, including MIC ranges, concentrations that inhibited 50% and 90% of the isolates tested (MIC_50_ and MIC_90_, respectively), geometric mean (GM) MICs, and modal MICs. Differences in SCY-247 plasma and tissue concentrations were assessed for significance by ANOVA with Dunnett’s post-test for multiple comparisons. Differences in fungal burden (CFU/g) were assessed for significance by the Kruskal-Wallis test with Dunn’s test for multiple comparisons. A *P*-value of <0.05 was considered statistically significant for all comparisons.
